# Errata

**Published:** 1782

**Authors:** 


					/>. to1?. Jo, far termined read termed ; p, log, /? ia,
{or empiric read empiric; p. Me^'i lb, [?r ?ue rWi fur j /. 20, f6r.
I lgc^lionfs read fngenhoulz ; p. 112, I- iz, fir fetoa read fet? ;

				

